# Milk Quality Conceptualization: A Systematic Review of Consumers’, Farmers’, and Processing Experts’ Views

**DOI:** 10.3390/foods12173215

**Published:** 2023-08-26

**Authors:** Greta Castellini, Serena Barello, Albino Claudio Bosio

**Affiliations:** 1 EngageMinds HUB—Consumer, Food & Health Engagement Research Center, Università Cattolica del Sacro Cuore, 20123 Milan, Italy; greta.castellini@unicatt.it (G.C.); claudio.bosio@unicatt.it (A.C.B.); 2 Faculty of Agriculture, Food and Environmental Sciences, Università Cattolica del Sacro Cuore, Via Bissolati, 74, 26100 Cremona, Italy; 3 Department of Psychology, Università Cattolica del Sacro Cuore, Largo Agostino Gemelli, 1, 20123 Milan, Italy; 4 Department of Brain and Behavioural Sciences, University of Pavia, Via Agostino Bassi, 21, 27100 Pavia, Italy; 5 IRCAF (Invernizzi Reference Center on Agri-Food), Campus Santa Monica, Via Bissolati, 74, 26100 Cremona, Italy

**Keywords:** milk quality, representations, citizen-consumer psychology, farmer, concept mapping, processing expert

## Abstract

Milk consumption has traditionally been recognized as a fundamental element of global dietary patterns due to its perceived nutritional advantages. Nonetheless, a substantial decrease in milk consumption has been identified within diverse populations in recent times. Specifically, consumers’ expectations and representations of milk quality have undergone notable transformations, contributing to the observed reduction in consumption. The objective of this systematic review was to conduct a comprehensive examination and categorization of the conceptual attributes associated with milk quality, considering the representations of citizen-consumers, farmers, and processing experts. This review was conducted following the Preferred Reporting Items for Systematic reviews and Meta-Analyses (PRISMA) guidelines. The titles and abstracts of 409 articles were screened, and 20 full-text articles were assessed for eligibility. The results demonstrate the existence of a dual articulation in the conceptual definition of milk quality. Farmers and processing experts exhibited a relatively similar representation of milk quality, focusing on technical indicators. In contrast, citizen-consumers held more simplistic and subjective concepts that are challenging to quantify. This study emphasized the critical need for establishing a platform for communication and knowledge exchange to foster shared representations and expectations regarding milk quality.

## 1. Introduction

Milk consumption has long been regarded as a fundamental element of global dietary patterns due to its perceived nutritional advantages. However, a notable decline in milk consumption has been observed across various populations in recent times [1]. More specifically, there has been a 2% decline in milk consumption in the EU between 2013 and 2018, and this decrease is expected to continue [2]. The reduction in milk purchases is particularly relevant in Italy, where its consumption has been decreasing in a progressive way, from 56.4 L per capita in 2009 to 50.2 L in 2014 (6%) [3]. Research indicates that this decline is influenced by several significant factors, with particular emphasis on the profound shifts in consumers’ perceptions of milk quality. These altered expectations and representations of milk quality have contributed to the observed consumption decrease [4]. Firstly, there has been a growing emphasis on health and nutrition as key dimensions of milk quality. Consumers are increasingly focused on nutritional content, the absence of harmful additives, and the overall health and environmental impact of the foods they consume. For instance, recent studies have demonstrated how health and animal welfare concerns can impact the hedonic and emotional response to milk and subsequently affect consumption [5]. Additionally, sustainability and ethical considerations have taken center stage as crucial aspects of food quality. Consumers now prioritize environmentally friendly production methods, fair trade practices, and animal welfare in their definitions of quality. These aspects seem to be particularly important for those countries that have limited natural resources and are densely populated [6]. Asian countries such as China and India are increasing the attention paid to food sustainability as they perceive the risk of not having enough resources to meet the needs of the entire population [6,7]. Sensory attributes and taste, though still crucial, are now being sought after for more diverse and authentic taste experiences, often linked to cultural preferences and personal enjoyment. A study conducted in Latin America (Mexico and Chile), Europe (Italy, Spain, Greece, and Denmark), and Asia (Bangladesh) showed that, in European and Asian countries, sheep and goat dairy products are not consumed because consumers dislike them, while in Mexico a higher percentage of people do not consume these dairy products because they are unfamiliar with them [8]. Moreover, convenience, affordability, and transparency in the food supply chain are emerging as significant factors shaping consumer perceptions of food quality, leading to profound shifts in how milk quality is defined.

From a legislative point of view, the rules introduced to protect the quality of milk are many and vary from country to country [9]. European Union regulations encompass a series of legislative measures that comprehensively cover various aspects of the dairy sector. The production of dairy products adheres to general hygiene prerequisites outlined in several European regulations: Regulation (EC) No 178/2002 [10], Regulation (EC) No 852/2004 [11], and Regulation (EC) No 853/2004 [12,13]. Processed milk must meet stringent hygiene criteria, including limits on microorganisms, somatic cell counts, the absence of veterinary drug residues, and not surpassing acceptable levels of specific contaminants. Moreover, compliance with public health standards is imperative. For instance, non-EU nations must possess an approved monitoring scheme for “residues”. Items introduced into the EU market must adhere to food law requisites, notably Regulation (EC) No 178/2002 [10]. The legislation also incorporates specifications for product labeling. Variations exist in standards and labels for milk fat and spread products across different global regions [14]. Several authors have assessed the implications of the new EU Regulation No. 1169/2011 [15,16]. Within the EU, Regulation No 931/2011 [10] pertaining to the traceability of animal products, Regulation No 1169/2011 [17] addressing consumer information provision, and Regulation No 1308/2013 [18] governing the organization of agricultural markets collectively serve as the principal legislative frameworks overseeing milk labeling.

However, current marketing strategies reveal a gap in adopting a comprehensive approach that considers the perspectives of both dairy experts and citizen-consumers regarding milk quality. This fragmentation in milk quality definitions has resulted in the formulation of marketing and communication strategies that have proven to be ineffective and unsuccessful, ultimately negatively impacting milk consumption [19]. Built upon these premises, it is imperative for the dairy industry to grasp and explore the societal perspective regarding milk quality as underscored by the Food and Agriculture Organization of the United Nations (FAO) [20]. Specifically, it is of utmost importance to investigate the novel representations and quality attributes of citizen-consumers pertaining to milk and ascertain whether these are aligned with those of experts such as farmers and processing experts. This comprehension plays a pivotal role in the development of products and the formulation of marketing strategies that cater to the ever-evolving needs and demands of consumers [2,21]. Notably, for citizen-consumers, it is crucial that certain characteristics of milk are visible and comprehensible in order to minimize uncertainty and prevent dissatisfaction.

However, the scope of research that focuses on the concept of milk quality beyond the existing technological and hygienic definitions remains limited [22]. While current knowledge about milk quality is valuable, it does not encompass all possible ways of representing and conceptualizing its meaning.

To bridge this knowledge gap, the objective of this systematic review was to undertake a comprehensive examination and categorization of the conceptual attributes associated with milk quality, considering the viewpoints of citizen-consumers, farmers, and processing experts.

The specific objectives of this review are as follows: (a) to identify the primary attributes that define milk quality, taking into account the perspectives and distinct representations of citizen-consumers, farmers, and dairy processing experts (advisors and processors); (b) to examine the differences and similarities in the representation of milk quality among these key stakeholders in the dairy industry; (c) to categorize these attributes of milk quality conceptualization utilizing an ecological framework to provide a comprehensive description and analysis.

## 2. Materials and Methods

This systematic review was conducted and reported following the Preferred Reporting Items for Systematic reviews and Meta-Analyses (PRISMA) guidelines [23].

### 2.1. Search Strategy

A comprehensive search strategy was formulated to identify relevant peer-reviewed publications pertaining to the determinants influencing the perception of milk quality among farmers, citizen-consumers, and processing experts (advisors and processors). In the context of the milk supply chain, farmers are individuals or entities primarily engaged in dairy farming. They manage farms where dairy animals, such as cows, goats, or sheep, are raised for the purpose of producing milk. Processors are entities responsible for collecting, pasteurizing, processing, and packaging milk. They play a vital role in ensuring that raw milk is transformed into a safe, shelf-stable, and consumer-ready product through processes that involve heat treatment, separation, and other techniques. [24]. The strategy employed a combination of keywords extracted from titles and abstracts. The search terms were grouped into three categories: (I) the concept of milk quality, which was searched as a single term while excluding closely related concepts to ensure conceptual clarity; (II) specific domains of interest such as perception, attitude, and expectation; and (III) the target subjects of interest, namely, farmers, citizen-consumers, and processing experts (identified as processors and advisors). The following search string was developed: (milk quality) and (acceptance*) OR (opinion*) OR (perception*) OR (attitud*) OR (evaluation) OR (valuation) OR (adopt*) OR (defin*) OR (expectation*) OR (determinant*) OR (criteri*) OR (factor*) OR (representation*) OR (attribute*) and (consumer*) OR (citizen*) OR (shopper*) OR (user*) OR (public) OR (buyer) OR (farmer∗) OR (processor∗) OR (stakeholder∗) OR (supply chain∗) OR (producer).

This search strategy was adapted to the thesaurus characteristics of each considered database (i.e., SCOPUS, PSYCINFO, WEB OF SCIENCE, and PUBMED) and launched in December 2022. Literature search was limited to peer-reviewed studies published in English or Italian. No time restriction was applied, so as to be as inclusive as possible. Reference lists of eligible studies and review articles were scanned to identify any missed articles.

### 2.2. Study Selection and Data Extraction

A three-step screening process was implemented to identify suitable studies for inclusion in this review, as described by [25]. In cases where there was disagreement between the two reviewers, all three researchers discussed the articles until a consensus was reached.

For all selected studies, the authors extracted information included study author(s), year of publication, countries where the study was carried out, sample characteristics (including sample size, age, and percentage of females involved), and study design. Moreover, the type of milk investigated and the type of participants (farmers, citizen-consumers, or processing experts) involved in the studies were extracted. In addition, attribute categories of milk quality were mapped for citizen-consumers, farmers, and processing experts (advisors and processors). Since the selected studies considered different attributes to define the concept of milk quality, they have been reviewed, selected, and grouped into macro-categories.

The data were extracted systematically using a standardized data extraction form as described by [25]. The extracted data were summarized in tables and a narrative synthesis was developed using a textual approach to synthesis the findings [26].

#### Procedure of Grouping Variables

**Figure 1:**
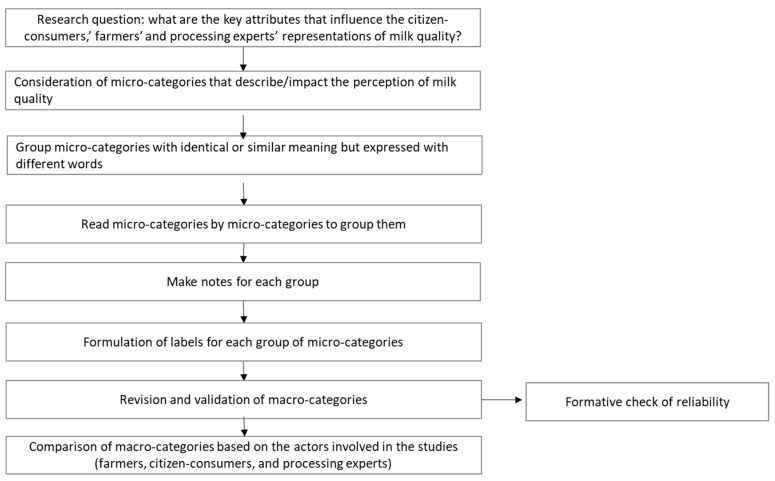
Procedure of inductive category development, adapted from Mayring [30] and Schilling [13].

The included studies reported several attributes (namely, “micro-categories”) to describe the concept of milk quality. These micro-categories were then grouped into broader macro-categories to allow for an effective synthesis of the results (Figure 1).

In particular, a qualitative content analysis procedure, widely implemented to analyze textual data [27], was adapted to reduce the number of categories. More specifically, conventional content analysis [27,28], also described as inductive category development [29], was applied because this procedure allows categories and their names to flow from the data instead of using preconceived categories [27]. The procedure for developing the categories of the extracted attributes is presented in Figure 1 and was carried out by three researchers independently (GC, SB and CB).

In order to handle the large amount of data, all the micro-categories that impact the concept of milk quality were transcribed into Excel. After that, the micro-categories were carefully re-read and those that referred to the same key concept were grouped under the same macro-category (e.g., all variables that mentioned worker hygiene, animal hygiene, or farm hygiene were grouped under the same macro-category), identifying labels that were consistent with the micro-categories grouped (e.g., hygiene quality).

Finally, the macro-categories were further validated (formative check of reliability) by the three researchers (GC, SB, and CB), checking the level of agreement among the categories created by the researchers independently and discussing cases of doubt and overlapping labels.

The validated macro-categories were used to compare differences and similarities among the different actors (citizen-consumers, farmers, and processing experts) with respect to the concept of milk quality.

### 2.3. Data Analysis

The macro-categories have been integrated and organized according to the framework of Story et al. [31] which is based on the socio-ecological framework of Bronfenbrenner and Capruso [32] and Bronfenbrenner [33]. This framework presents four systems within which people act, and these systems can be paramount in influencing the formation of one’s own opinions about social phenomena: (I) The Individual system, identified as the place where people generate opinions based on their experiences with the phenomenon (e.g., attributes related to milk-related sensory aspects); (II) The Microsystem, which is the context where opinions are structured and formed through comparisons with others (e.g., attributes related to the concept of trust towards milk producers); (III) The Mesosystem, which is the context where one’s own opinions are shaped by considering the tangible features of a phenomenon (e.g., milk’s nutritional value on the label, packaging features) or context (e.g., milk-related hygiene conditions, technological systems); and finally, (IV) the Macrosystem, which relates to the context of social norms (e.g., attributes concerning the legislative or policy systems related to milk). Subsequently, a comprehensive diagram was created to offer a visual depiction of the outcomes. This diagram encompassed the macro-categories linked to the concept of milk quality, which were subsequently classified and distinguished in alignment with the four systems of the socio-ecological framework. Moreover, the diagram portrayed the percentage distribution of micro-categories within each macro-category and system. Furthermore, the diagram facilitated a comparative analysis of the macro-categories, accentuating the distinctions and similarities among citizen-consumers, farmers, and processing experts (advisors and processors).

## 3. Results

### 3.1. Search Results

**Figure 2:**
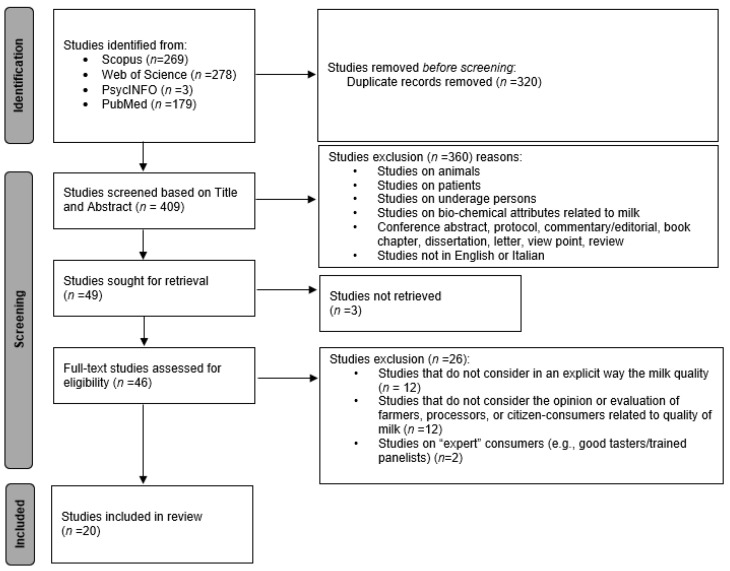
PRISMA flow diagram of study selection.

A total of 729 records were retrieved. A first screening round was conducted, eliminating 320 duplicate records. A further round of screening was applied to the title and abstracts on the remaining 409 records. After applying the eligibility criteria, 49 records were judged as potentially relevant. Another screening phase was applied to the remaining full-text articles to exclude articles not in line with the study’s objectives. Finally, according to the pre-defined eligibility criteria, 20 studies were identified as coherent with the inclusion/exclusion criteria as they focused on the attributes of the milk quality concept. Figure 2 describes the selection and screening process.

### 3.2. Studies’ Overview

**Figure 3:**
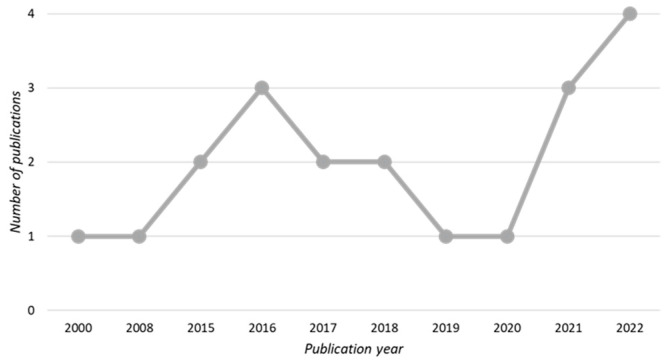
Time distribution of papers on milk quality.

**Figure 4:**
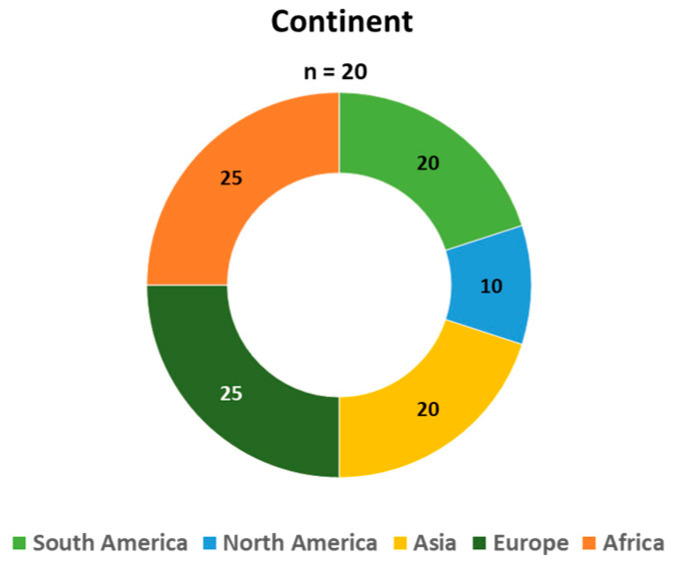
Geographical distribution of papers.

**Figure 5:**
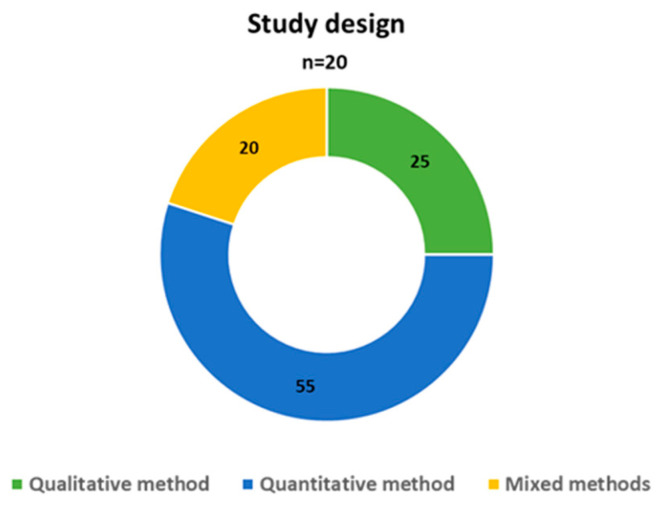
Study design of papers.

Table 1 provides an overview of the studies included in this analysis. The publications spanned from 2000 to 2022, with an increase in the number of studies observed in the recent years (2021–2022), as shown in Figure 3. Geographically, most studies were conducted in the Americas, including South America (*n* = 4, 20%) and the North America (*n* = 2, 10%), followed by Europe (*n* = 5, 25%), Africa (n = 5, 25%), and Asia (*n* = 4, 20%), as illustrated in Figure 4. Quantitative research designs were predominantly utilized in most studies (*n* = 11, 55%), as indicated in Figure 5. The sample sizes across the studies varied from *n* = 40 to *n* = 1646, as detailed in Table 1. The focus of nearly all studies was on cow’s milk quality (*n* = 20, 95%), with a significant involvement of farmers (*n* = 13, 65%). When considering the study design and participants involved, recent research conducted in 2021–2022 primarily employed qualitative methods (5 out of 7, 71%) and focused on the perspective of citizen-consumers (4 out of 7, 57%), while earlier studies conducted from 2000 to 2020 predominantly employed quantitative designs (9 out of 13, 69%) and mainly focused on the viewpoint of farmers (8 out of 13, 62%).

### 3.3. Attributes and Macro-Categories Related to the Concept of Milk Quality

**Figure 6:**
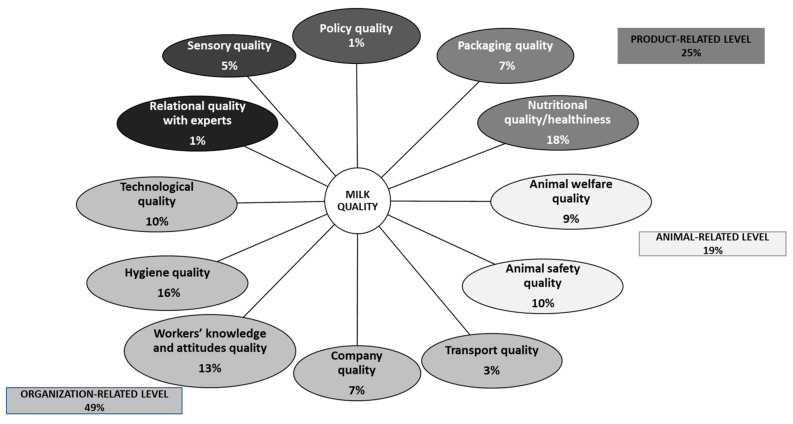
Macro-categories related to the concept of milk quality.

A total of 70 attributes (micro-categories) of milk quality were identified (see Supplementary Table S1). By employing the procedure of inductive category development adapted from Mayring [30], these 70 micro-categories were grouped into 12 macro-categories (Figure 6). Specifically, the results showed that the concept of milk quality is related to the following: (I) policy quality (i.e., transparency of the regulations ruling milk production and processing); (II) relation quality with expert (i.e., trust that people have in the producers and distributors of milk); (III) sensory quality (i.e., perceived organoleptic properties of milk); (IV) packaging quality (i.e., clarity and comprehensiveness of information on the milk pack); (V) nutritional quality/healthiness (i.e., nutritional value/perceived healthiness of milk); (VI) animal welfare quality (i.e., animal welfare protection); (VII) animal safety quality (i.e., animal health protection); (VIII) transport quality (i.e., speed and safety of product transportation/distribution); (IX) company quality (i.e., company reputation); (X) workers’ knowledge and attitudes quality (i.e., knowledge and experience of the producing company’s workers); (XI) hygiene quality (i.e., product hygiene protection); and (XII) technological quality (i.e., level of technological advancement of the producing company).

Some of these macro-categories are linked together by overarching dimensions. In particular, the macro-categories “packaging quality” and “nutritional quality/healthiness” refer to milk quality attributes related to the product; the macro-categories “animal welfare quality” and “animal safety quality” concern attributes related to animals; and “technological quality”, “hygiene quality”, “workers’ knowledge and attitudes quality”, “company quality”, “transport quality” are attributes related to the organizational context in which milk is produced or processed. Most of the micro-categories that connote the concept of milk quality are attributes related to the organization level (49%) and the product level (25%). In particular, the nutritional quality/healthiness (18%), hygiene quality (16%), and workers’ knowledge and attitudes quality (13%) are the most salient attributes in defining the concept of milk quality (Figure 6). Moreover, the results of this study showed that sustainability and in particular welfare and health of animals are becoming paramount aspects in defining quality in milk. Indeed, 19 % of the micro-categories analyzed considered this issue.

### 3.4. Classification of Micro- and Macro-Categories about the Concept of Milk Quality According to Bronfenbrenner’s Socio-Ecological Framework

**Figure 7:**
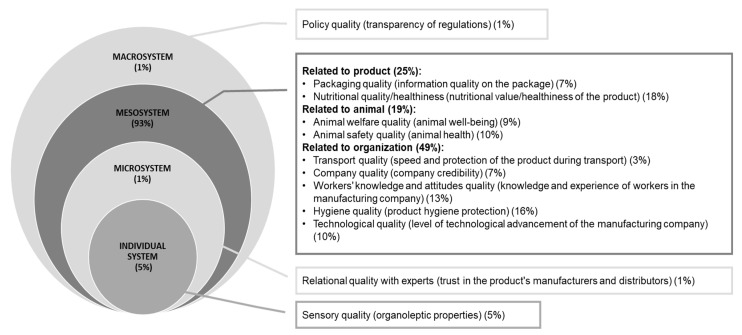
Classification of micro- and macro-categories regarding the concept of milk quality.

In accordance with Bronfenbrenner’s socio-ecological framework (1979), a significant proportion of macro-categories and their corresponding micro-categories associated with the concept of milk quality are situated within the Mesosystem (93%) (Figure 7). These findings highlight the predominant influence of beliefs and perceptions concerning the physical environment where milk is produced and processed on the understanding of milk quality. Conversely, less emphasis is placed on individual factors such as personal inclinations or taste preferences (Individual system; 5%), social norms encompassing trust and social influence (Microsystem; 1%), and cultural norms and agricultural policies (Macrosystem; 1%).

### 3.5. Milk Quality through the Lens of Citizen-Consumers, Farmers, and Processing Experts (Advisors and Processors)

**Figure 8:**
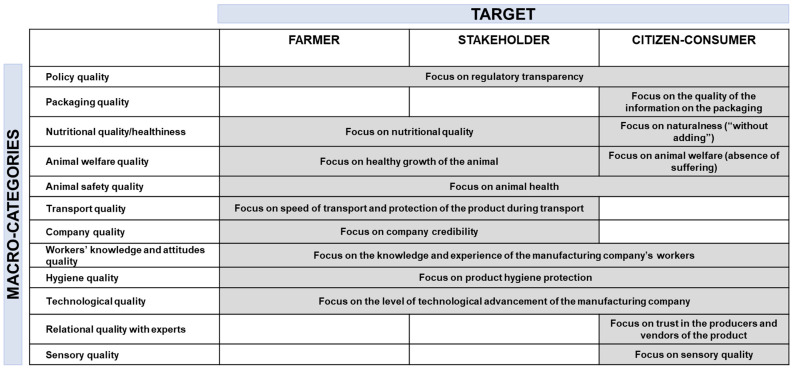
Farmer, processing experts, and citizen-consumer thematic content related to milk quality.

In this section, we describe the semantic attributes associated with the representations of the three main targets examined in this study. Figure 8 provides a detailed analysis of the main overlaps and thematic content concerning the conceptualizations of milk quality among these three representations.

Regarding the overlapping thematic content, our literature analysis reveals that several attributes of milk quality are relevant across different actors. For citizen-consumers, farmers, and processing experts (advisors and processors), milk quality is linked to transparency in regulations regarding milk quality requirements, production, and distribution processes. Additionally, all actors highlight the importance of an approach to milk quality and safety that ensures integrity from farm to glass. Furthermore, the results suggest a need to enhance farmers’ knowledge and attitudes and implement hygienic control in the milk production process to meet the required milk quality and food safety standards. Moreover, the conceptualization of milk quality appears to be influenced by the level of technological advancement of the production company. The more a company adopts innovation to ensure a high-quality chain from farm to glass, the more the milk is perceived as a quality product. Finally, citizen-consumers, farmers, and processing experts (advisors and processors) converge in defining milk quality as a product that guarantees certified animal health protection and exhibits high nutritional quality. However, the content of these attributes/themes related to milk quality varies among actors (Figure 8). Dairy experts (farmers and processing experts) assert that milk can be considered a quality product if animal welfare is upheld, including proper disease identification, milk culturing for pathogen detection, appropriate treatment options, and effective management techniques to reduce mastitis incidence. On the other hand, citizen-consumers contend that milk is of good quality when animals have not suffered and continue to live according to their natural behaviors (e.g., grazing, eating grass). Furthermore, while experts (farmers and processing experts) associate high nutritional value with milk quality based on its energy content, protein source, and calcium content, citizen-consumers perceive milk quality as determined by the absence of added ingredients and the naturalness of the product.

This duality in thematic and content perspectives characterizing the representations of milk quality by dairy experts and citizen-consumers, particularly regarding nutritional aspects and animal welfare, highlights how the former prioritize technical aspects such as animal diseases and somatic cell counts, whereas the latter hold simpler and more naïve concepts (e.g., absence of animal suffering or “free-from” products) in their representation of quality milk. Additionally, certain conceptual attributes of the milk quality definition appear to be target-specific. For example, only citizen-consumers identify clear and transparent labels related to nutritional properties, trust in dairy experts, and organoleptic qualities (e.g., appearance, taste, smell) as attributes of milk quality. Conversely, farmers and processing experts (advisors and processors) share similar perspectives on the definition of milk quality, emphasizing two attributes: the speed and protection of milk during transportation from the farm to the industry and the credibility of the production company.

## 4. Discussion

The decrease in milk consumption can be attributed to several multifaceted factors, among which the evolving notion of milk quality among citizen-consumers plays a pivotal role in contributing to this decline [4]. To address this concern, it is essential to understand how citizen-consumers perceive milk quality and ascertain whether their perception aligns with that of experts, including processing experts and farmers. As a result, we undertook a systematic review with the objective of identifying the crucial attributes that shape the concept of milk quality across three key stakeholder groups: farmers, processing experts (advisors and processors), and citizen-consumers.

The findings reveal that, while milk quality is a relatively new research area, there has been a notable surge in studies conducted on this topic in recent years (2021–2022). Additionally, recent studies have predominantly adopted qualitative methodologies, focusing on the perspective of citizen-consumers, in contrast to earlier research trends. This shift can be attributed to evolving consumer demands, which have significantly reshaped the broader notion of food quality [54]. Currently, food quality is not solely linked to functional parameters like nutritional value, appearance, and taste; it is also deeply intertwined with the ethical, identity, and emotional values of citizen-consumers [55,56]. Furthermore, in terms of the geographical distribution of the studies, the results indicate a heightened interest in the subject of milk quality in Africa and Asia. This observation could be attributed to the necessity of these regions to enhance and promote high-protein foods as a strategy to address malnutrition rates [57], where milk emerges as a potential key solution [58].

The findings show the presence of 12 main attributes (macro-categories) that characterize the concept of milk quality. Many of these attributes pertain to the organizational and product levels. Notably, nutritional quality/healthiness, hygiene quality, and workers’ knowledge and attitudes emerge as the most prominent attributes in defining the concept of milk quality. These findings are consistent with previous research indicating that milk quality is primarily associated with its nutritional and hygienic aspects [59] and the skills of workers, which significantly impact the economic efficiency of dairy farms [60]. In particular, hygiene standards are defined and regulated differently depending on the country of reference. As for the nations belonging to the European Union, appendix III, section IX, chapter I of Regulation (EC) No 853/2004 of the European Parliament [11] and of the Council of 29 April 2004 [12] describe the acceptable numbers of bacterial and somatic cells in milk to define it as safe and therefore saleable. However, in some countries outside the European Union, food safety legislation is poor, resulting in scarce hygienic practices in the treatment of milk. In the South African territories, for example, the lack of regulation with respect to hygienic standards in the treatment and sale of milk is considered the main reason for losses, resulting in reduced income for the farmers and for the smallholder dairies [61]. Also, in Ethiopia, there is no hygiene standard followed by producers during milk production. Hygiene conditions vary depending on the production system. In most cases, under small-scale farming conditions, the common hygiene measures adopted during milk production, especially during milking, are limited to allowing the calf to suckle for a few minutes and/or washing the udder before milking [62]. However, the aspect of sustainability, particularly animal welfare and health, is increasingly recognized as a crucial component in defining quality milk. In line with this, several studies have highlighted that controlling cow mastitis and somatic cell count (SCC) is a significant concern for farmers in maintaining milk quality [63]. Furthermore, there are some studies that claim farmers are very attentive to the animals’ diets, as they are aware that it impacts the features of milk [64,65]. For instance, it has been demonstrated that pasture feeding positively influences the nutritional profile of milk, enhancing its health benefits [66], which is highly valued by consumers. Additionally, the animals’ diets affect the organoleptic qualities of milk [67]. For example, the ratio between maize silage and lucerne silage can impact the milk’s color, creaminess, and density, indirectly influencing quality assessment [68]. Moreover, the environmental and welfare conditions to which cows are exposed can influence the organoleptic and nutritional characteristics of milk [69]. Specifically, subjecting cows to significant stress due to poor welfare conditions results in decreased milk production with lower levels of fat and protein, thus rendering the milk less nutritious and of inferior quality [70]. Finally, paying attention to the well-being and health of animals is not only important for producing quality milk, but also for achieving a positive economic return. In fact, dirty and poorly maintained environments can increase the likelihood of animals getting sick and requiring antibiotic treatments, resulting in additional and often prohibitive costs for the farmers [71]. Considering citizen-consumers, recent research indicates that they associate milk quality with factors such as free cow grazing, natural feed, and the absence of medical treatment for cows [72,73]. Moreover, technological development and automation of breeding and milking processes are relevant in defining milk quality, as shown by past studies [74]. However, it is interesting to note that traceability technologies are not mentioned. This aspect points out that, although such technologies have been implemented to increase milk controls in order to ensure a quality product [75], these, in the imaginations of the targets considered, are not linked to the attributes of milk quality. Supporting these findings, some studies showed that perceptions of and interest in traceability change across countries [76]. Although in most cases traceability is strongly perceived as synonymous with genuine and safe product, those who do not trust certifying bodies, technology, and have little knowledge do not consider them as part of the safety- and quality-assurance strategy in the food industry [77].

Regarding the classification of micro- and macro-categories within Bronfenbrenner’s socio-ecological framework, it is evident that attributes related to taste preference received minimal mention from the study participants. These findings appear to contradict previous research, which commonly associates food quality with personal evaluations based on taste and liking. However, the attributes utilized by individuals to describe quality are dynamic and subject to change based on their interests, concerns, or needs [4]. Several studies [4,21,78] have observed a recent shift wherein extrinsic quality attributes, which pertain to characteristics associated with a product but are not physically inherent to it [79], have gained increasing importance in defining food quality, alongside intrinsic attributes, which are related to the physical composition of the product itself and cannot be altered without changing its nature, such as aroma, taste, and color [79,80]. Of particular significance are the extrinsic quality attributes known as “Search Qualities,” which individuals can determine before purchasing a food product through direct examination (e.g., nutritional value or packaging size and features), and “Credence Qualities” [81], which require additional information for evaluation and cannot be directly experienced from the product itself (e.g., environmental impact). These extrinsic qualities have gained importance as sustainability and company practices have become priorities in food choices [82]. For example, many quality food characteristics have been associated with farming practices and the entire distribution chain, including the processes from farm to fork and how crops and livestock are managed [4,83,84]. Therefore, the extrinsic quality aspects related to product features and the physical environment in which food is processed and produced are the most utilized attributes in defining food product quality, as affirmed by the present study. In summary, it can be concluded that the perceived quality of milk is primarily shaped by extrinsic attributes associated with the production and processing of milk, while intrinsic attributes tied to individual sensory perceptions appear to have less prominence in the representation of milk quality. Lastly, the attributes employed to define the concept of milk quality vary among the study participants. Farmers and processing experts appear to share a relatively similar perception of milk quality, marked by technical indicators and a strong emphasis on knowledge and expertise. In contrast, citizen-consumers hold a representation of quality milk rooted in simplified and less sophisticated concepts (such as the absence of animal suffering or “free-from” products). For example, low-fat milk, milk without additives, and milk derived from animals not treated with antibiotics are among the aspects that consumers pay attention to [41,85]. These aspects can be challenging to quantify and are primarily tied to their individual perceptions, which may not always be based on concrete evidence. Previous research has highlighted citizen-consumers’ concerns regarding farming practices that they believe impact the emotional well-being of animals, the treatment of animals, and the idea of naturalness [86]. Furthermore, even when various stakeholders share a common conceptual category for defining milk quality (such as “animal welfare”), they diverge in the interpretations assigned to it (like “physical health of the animal” versus “well-being and safeguarding of the animal’s quality of life”). This could suggest only an apparent alignment of perspectives, but it reveals a profound fragmentation of the semantic framework within which the representations of quality milk are generated by the different social actors involved in the milk production and consumption arena. From our standpoint, this study offers valuable insights for future research in the field. Primarily, it underscores the necessity to delve deeper into the fundamental attributes that shape the concept of milk quality through both qualitative and quantitative investigations. This endeavor will contribute to a more comprehensive grasp of the representations held by various stakeholders, encompassing both experts and non-experts within the dairy industry, especially given the notable disparities revealed in this study. Moreover, it is imperative for scholarly experts in the dairy domain to adopt a more holistic research approach when addressing these matters. Moving away from a self-referential perspective, an interdisciplinary approach should be embraced to scrutinize the concept of milk quality. This approach should encompass an ecological perspective that integrates a variety of disciplines, aiming to present a more cohesive portrayal of milk quality. Furthermore, this perspective should be reassessed and harmonized through a bottom-up strategy in conjunction with the viewpoint of citizen-consumers. To facilitate this, initiatives that facilitate dialogue and collaboration between citizens and industry experts, such as participatory and citizen science methods, should be encouraged. These initiatives will serve to educate and involve citizen-consumers in conversations about milk quality, ultimately fostering the development of a shared comprehension and addressing the dual fragmentation present between the interpretations of milk quality among dairy experts and citizen-consumers. Moreover, in order to have a more complete view related to the “milk quality” topic, it might be interesting to conduct new research involving other supply chain actors not included in this study, such as sellers. Lastly, it is also crucial to investigate spontaneous discourse and social communication related to milk quality. This analysis will enable a deeper insight into the ongoing conversations surrounding this subject and yield valuable concepts and perspectives for further exploration in this field.

## 5. Conclusions

This systematic review marks the inaugural scientific effort dedicated to exploring the psychosocial discourse surrounding milk quality as documented in the literature, yielding promising outcomes. Specifically, the study delved into the core attributes associated with the concept of milk quality across three key stakeholders: citizen-consumers, farmers, and processing experts (including advisors and processors). The findings unveil that the definition of milk quality revolves around 12 major conceptual categories, which can be organized within the framework of Bronfenbrenner’s ecological theory into four distinct systems. Notably, the representation of milk quality exhibits significant variation among the three targeted groups, particularly between expert figures in the dairy system (processing experts and farmers) and citizen-consumers. The study outcomes contribute to establishing a more methodical comprehension of the representations connected to the concept of milk quality, as perceived by all social actors involved in its production and consumption. Moreover, the findings underscore the necessity of fostering transdisciplinary and cross-sectoral links among perspectives stemming from diverse paradigms. Furthermore, the results underscore the importance of instigating a collaborative process to construct a shared social representation on this topic that effectively merges societal impact with a solid scientific foundation. To bridge the gap in perception and align milk quality representation, several educational strategies can be employed. For example, the experts (farmers and processors) can be encouraged to provide consumers with insights into their farming/production practices. This could involve hosting farm tours, workshops, or online videos that showcase the daily routines, animal welfare standards, and quality control measures undertaken on the farm and in the company. Moreover, organizing workshops for both experts and consumers can serve as a platform for knowledge exchange. Experts can gain insights into consumer preferences and concerns, while consumers can learn about the complexities of milk production. This two-way dialogue can bridge understanding and highlight the efforts that farmers and processors put into ensuring quality. Finally, introducing educational programs in schools that highlight the journey of milk from farm to table can cultivate informed consumer choices from a young age. Engaging activities, like farm visits or virtual tours, can make the learning experience more interactive and memorable. By implementing these educational strategies, farmers, processors, and consumers can collaborate to build a shared perception of milk quality. These efforts will not only foster transparency and trust but also contribute to the sustainability of the dairy industry by ensuring that products meet the expectations of both experts and consumers.

## Figures and Tables

**Table 1:** General features of included studies.

Study	Country	Study Design	Sample Size	Age Range (Mean Years, SD)	Gender (% Female)	Type of Milk	Point of View
[34]	USA	Qualitative research	93	18–25 (20, NR)	41%	Cow milk	Lay citizens
[35]	India	Mixed methods (qualitative and quantitative)	120	NR	NR	Cow milk	Farmers Intermediaries Retailers Traditional processors Consumers Key informants from government regulatory bodies Private and non-profit sectors
[36]	Mexico	Quantitative research	40	NR (52.65 ± 12.15)	NR	Cow milk	Farmers
[37]	Tanzania	Mixed methods (qualitative and quantitative)	208	NR	NR	Cow milk	Farmers Intermediaries Vendors Consumers Government officials Private sector donors
[38]	France	Participative approach (focus group meetings/Delphi)	44	N.R.	N.R.	Cow milk	Processor
[39]	Kenya	Mixed methods (qualitative and quantitative)	723	Most of the respondents were aged between 30–60 years	50%	Cow milk	Farmers
[40]	Italy	Quantitative research	1216	Most of the respondents were aged <65 years	68%	Cow milk	Lay citizens
[41]	India	Quantitative research	300	19–76 (40, NR)	3%	Cow milk	Farmers
[42]	Brazil	Qualitative research	557	>18 years old	35%	Cow milk	Dairy farmers Agricultural advisors Lay citizens
[43]	USA	Quantitative research	217	NR	NR	Cow milk	Farmers
[44]	Zimbabwe	Quantitative research	344	Most of the respondents were aged >30 years	NR	Cow milk	Farmers
[45]	Brazil	Mixed methods (quantitative and qualitative)	336	>18 years old, most of the respondents were aged 25–34	54%	Cow milk	Lay citizens
[46]	Colombia	Quantitative research	46	NR	NR	Cow milk	Farmers
[47]	Germany	Quantitative research	1646	>18 years old, the majority of the respondents were aged >60 (32%)	18%	Cow milk	Lay citizens
[48]	Ethiopia	Quantitative research	160	NR (42.14;14.50)	32%	Cow milk	Lay citizens
[49]	Ireland	Participative approach (focus group meetings/Delphi)	112	NR	NR	Cow milk	Farmers Stakeholders
[50]	Tanzania	Quantitative research	105	>18 years old, most of the respondents were aged <45 (83%)	46%	Cow milk	Dairy farmers Milk vendors Milk retailers
[51]	Indonesia	Quantitative research	33	NR	NR	Goat milk	Farmers Lay citizens
[52]	Denmark and Netherlands	Qualitative research (focus group approach)	25	30–60 (NR, NR)	NR	Cow milk	Farmers Advisors
[53]	Indonesia	Quantitative research	1225	NR	NR	Cow milk	Lay citizens

Note: NR = Note Reported, SD = standard deviation.

## Data Availability

Data are fully available upon request to the corresponding author.

## Supplementary Materials

The following supporting information can be downloaded at: https://www.mdpi.com/article/10.3390/foods12173215/s1, Table S1. Micro and Macro categories about milk quality representation.
