# Supplementary material for: Milk Quality Conceptualization: A Systematic Review of Consumers’, Farmers’, and Processing Experts’ Views
Source: Foods. 2023 Aug 26;12(17):3215. doi: 10.3390/foods12173215 (PMC10486532; doi:10.3390/foods12173215)
Supplement: Supplementary file 1 [file foods-12-03215-s001.zip › foods-2573587-SI.pdf]

Supplementary Materials

Table S1. Micro and Macro categories about milk quality representation

Macro-Categories	Micro-categories
**Sensory quality**	particle size
	milk color
	taste
**Relational quality with experts**	trust the retailer/vendors
**Animal welfare quality**	animal space
	animal growth
	animal nutrition/feed quality/natural feed
	grazing
	affective states related to animals
	natural life of animals
**Animal safety quality**	mastitis
	animal health
	helminthosis
	trypanosomiasis
	respiratory conditions
	tick-borne diseases
	udder disease
**Technological quality**	technological production techniques
	thermal stability
	suitability for preservation
	not fertilization method (ai)
	automatic milking systems (ams)
	quality of equipment/tools
	adequate cooling facilities
**Nutritional quality/healthiness**	lipid effects/fat content
	nitrogen effects
	plant components effects
	carbohydrate effects
	protein inputs
	rejection the use of antibiotics, hormones, and pesticides, transgenic components
	mineral inputs
	vitamins inputs
	organic milk
	nutritional content/value
	anything added to it
	unpasteurized milk
	local product
**Hygiene quality**	hygiene of livestock housing
	hygiene of environment (farm)
	hygienic practices in handling milk
	hygienic storage practices
	cleanliness of contenitors
	personal hygiene/hand washing
	cleanliness of milking area
	storage conditions
	colony-forming units (cfu)

	somatic cell count (scc)
	the cleanliness of the retail outlet
**Workers' knowledge and attitudes quality**	trained workers
	knowledge of milking practices
	knowledge on cleanliness of the mammary gland prior to milking
	knowledge of proper conduction of cows to the milking site
	milkers' motivation (good attitude towards their jobs)
	knowledge of milk quality regulations/parameters
	knowledge parameters and tests used by milk buyers,
	good attitudes towards milk quality requirements
	knowledge about the penalties for not producing quality milk
**Company quality**	family farmers
	small farm
	breed quality
	good work organization
	the competitive forces of the agribusiness.
**Packing quality**	term fresh/freshness on pack
	data expiration
	packaging (size, shape)
	nutritional information on label
	brand
**Transport quality**	selling milk quickly
	distances and time to reach the processor
**Policy quality**	legislation
